# Incorporating alignment uncertainty into Felsenstein’s phylogenetic bootstrap to improve its reliability

**DOI:** 10.1093/bioinformatics/btz082

**Published:** 2019-02-06

**Authors:** Jia-Ming Chang, Evan W Floden, Javier Herrero, Olivier Gascuel, Paolo Di Tommaso, Cedric Notredame

**Affiliations:** European Molecular Biology Laboratory, European Bioinformatics Institute, Wellcome Genome Campus, Cambridge CB10 1SD, UK; Centre for Genomic Regulation (CRG), The Barcelona Institute of Science and Technology, Barcelona 08003, Spain; European Molecular Biology Laboratory, European Bioinformatics Institute, Wellcome Genome Campus, Cambridge CB10 1SD, UK; Unité Bioinformatique Evolutive, Institut Pasteur, C3BI USR 3756 IP CNRS, Paris 75015, France; Centre for Genomic Regulation (CRG), The Barcelona Institute of Science and Technology, Barcelona 08003, Spain; Centre for Genomic Regulation (CRG), The Barcelona Institute of Science and Technology, Barcelona 08003, Spain; Universitat Pompeu Fabra (UPF), Barcelona 08003, Spain

## Abstract

**Motivation:**

Most evolutionary analyses are based on pre-estimated multiple sequence alignment. Wong *et al.* established the existence of an uncertainty induced by multiple sequence alignment when reconstructing phylogenies. They were able to show that in many cases different aligners produce different phylogenies, with no simple objective criterion sufficient to distinguish among these alternatives.

**Results:**

We demonstrate that incorporating MSA induced uncertainty into bootstrap sampling can significantly increase correlation between clade correctness and its corresponding bootstrap value. Our procedure involves concatenating several alternative multiple sequence alignments of the same sequences, produced using different commonly used aligners. We then draw bootstrap replicates while favoring columns of the more unique aligner among the concatenated aligners. We named this concatenation and bootstrapping method, Weighted Partial Super Bootstrap (wpSBOOT). We show on three simulated datasets of 16, 32 and 64 tips that our method improves the predictive power of bootstrap values. We also used as a benchmark an empirical collection of 853 one to one orthologous genes from seven yeast species and found wpSBOOT to significantly improve discrimination capacity between topologically correct and incorrect trees. Bootstrap values of wpSBOOT are comparable to similar readouts estimated using a single method. However, for reduced trees by 50 and 95% bootstrap thresholds, wpSBOOT comes out the lowest Type I error (less FP).

**Availability and implementation:**

The automated generation of replicates has been implemented in the T-Coffee package, which is available as open source freeware available from www.tcoffee.org.

**Supplementary information:**

Supplementary data are available at *Bioinformatics* online.

## 1 Introduction

Phylogenetic reconstruction tools are among the most widely used computational methods in biology (Guindon *et al.*, 2010; Kumar *et al.*, 2018; Stamatakis, 2006) with a wide range of applications ranging from epidemiology (Romero-Severson *et al.*, 2016; Zhukova *et al.*, 2017), ecology (Gascuel *et al.*, 2015), functional genomics (Ullah *et al.*, 2015), regulatory network evolutionary analysis (Brawand *et al.*, 2011) to protein structure comparison (Magis *et al.*, 2010). The availability of an increasing amount of sequence data, obtained by high throughput sequencing, is rapidly amplifying this trend (Rokas *et al.*, 2003). Nonetheless, correctly estimating phylogenetic trees remains a challenging task from both computational and biological standpoints.

A phylogenetic tree is the binary representation of an evolutionary scenario where each node represents either a duplication or a speciation event. Phylogeny has the aim of reconstructing the scenario best supported by variations measured across homologous sequences. Given the alignment of the set sequences, the task of estimating hidden mutations and searching the most likely tree is NP-Complete under its most common formulations [e.g. Maximum parsimony (Graham and Foulds, 1982)].

Computing accurate Multiple Sequence Alignments (MSAs) comes, however, with issues of its own (Chatzou *et al.*, 2016). Not being able to define and estimate a correct MSA unambiguously is a major problem when doing phylogenetic reconstruction. Wong *et al.* have previously exposed a few practical consequences of this situation when reconstructing phylogenetic trees (Wong *et al.*, 2008). They have shown that given a collection of 1502 datasets of homologous sequences, the seven most widely used aligners result in unexpectedly diverse phylogenetic trees with 46% of the datasets associated with more than one topology. Trees are even unstable in large scale data and their topology is affected by only changing sequence input order. Chatzou *et al.* have shown over 38% of branches are sensitive to the sequence input order in the dataset of HOMFAM with larger than 100 sequences (Chatzou *et al.*, 2018).

Until recently, this problem had been all but ignored by the community, with the vast majority of published trees relying on a single aligner, frequently ClustalW. When doing so, the issue of MSA reliability is usually addressed using a post-processing method, such as column trimming, in order to systematically remove the portions of an MSA unlikely to be correct (Capella-Gutiérrez *et al.*, 2009; Castresana, 2000). However recent results suggest trimming to have only a limited impact on phylogenetic estimation (Dessimoz and Gil, 2010; Liu *et al.*, 2009; Saurabh *et al.*, 2012). Moreover, it has been shown that excessive trimming can worsen the inferred phylogenetic tree (Tan *et al.*, 2015). Some protocols address this problem through systematic MSA sampling. For instance, the Heads-or-Tails procedure involves aligning the reversed sequences and comparing the direct and reverse versions of the MSA (Landan and Graur, 2007, 2008). This makes it the equivalent of a two-replicates sampling strategy. PRANK employs a more sophisticated approach with each MSA replicate estimated by randomly breaking all dynamic programing ties (Löytynoja and Goldman, 2008). The most elaborate (and time intensive) protocol is probably GUIDANCE where MSA replicates are obtained by re-estimating progressive MSAs using guide trees obtained from the bootstrap replicates of the default MSA (Penn *et al.*, 2010a, b). A slightly more general approach was recently described (Chang *et al.*, 2014, 2015), based on the T-Coffee consistency framework. It involves estimating the local reliability of an existing MSA using a library of pairwise alignments. All these sampling strategies produce comparable output, in the form of an index summarizing the alignment robustness of each residue across the MSA sampling process.

Various benchmarks, by others and us, have shown these indexes to be very informative as accuracy estimators, and comparable in their specificity (Chang *et al.*, 2014; Landan and Graur, 2007; Penn *et al.*, 2010a). These estimates of MSA accuracy address the uncertainty issue raised by Wong *et al.* but they stop short of providing a definite answer to the effect of MSA uncertainty on phylogenetic reconstruction, especially when dealing with non-controlled cases. If a dataset is so complex that seven aligners can produce seven MSAs different enough to support more than a single tree, one needs to know how such variation affects phylogenetic reconstruction.

In this work, we are proposing a bootstrap method that precisely addresses this issue. We show that rather than combining the alternative MSAs into a unique consensus model or locally trimming them on the basis of their agreement, one can concatenate those alternative MSAs into a Super-MSA which is used to infer an tree. Then, the tree’s confidence is estimated by using replicates drawn from the Super-MSA. Doing so results in bootstrap values that simultaneously reflect site sampling (as regular bootstrap) along with the MSA induced uncertainty. While this procedure does not significantly improve the accuracy of the inferred tree, it makes the global bootstrap index more informative in identifying problematic branches or trees. Recently, Ashkenazy *et al.* have shown tree topology accuracy can be improved through concatenating those alternative alignments into a single Super-MSA (Ashkenazy *et al.*, 2018). Interestingly, we came out the same idea in parallel with Ashkenazy *et al.* in concatenation of MSAs even with almost identical naming. However, we aim to investigate the benefit of incorporation MSA variation into bootstrap instead of boosting overall tree accuracy.

## 2 Materials and methods

Bootstrap allows estimating the tree topology stability or the support for a clade based on random sampling with replacement. There are two key points: what are measures (Section 2.1) and how random sampling is performed (Section 2.2). We revised the second part by developing alternative sampling protocols for incorporating alignment uncertainty. Original bootstrap support methods estimate tree topology stability by randomly drawing columns from an original MSA (replicates). Our approach is similar but involves drawing the columns from a collection of alternative MSAs rather than a single MSA. When doing so, support eventually reflects both the site sampling process (standard bootstrapping measures) and also the consistency across MSAs. If the alternative MSAs are identical, the support remains the same but decreases if MSAs are diverse. This section is organized as the following: first, we define bootstrap measures in the Section 2.1. Then, the detailed sampling is described in Section 2.2. Aligners’ use for the comparison is covered in Section 2.3. Two benchmarks are used for validation based on simulated and empirical data in Sections 2.4 and 2.5, respectively. Finally, we introduce evaluation metric in Sections 2.6 and 2.7.

### 2.1 Bootstrap measures

**Fig. 1.**
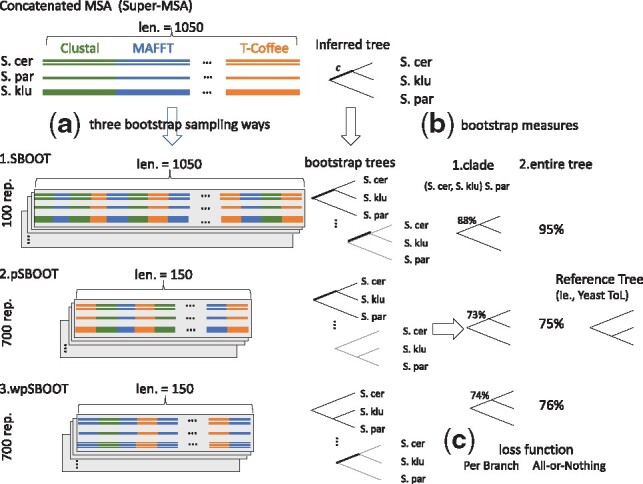
Flowchart of Super-MSA alternative sample methods. (**a**) We proposed three sampling ways, super bootstrap (SBOOT), partial super bootstrap (pSBOOT) and weighted partial super bootstrap (wpSBOOT). *SBOOT*: applying the standard bootstrap on the concatenated MSA. *pSBOOT*: partial sampling on the concatenated MSA. *wpSBOOT*: weighted partial sampling on the concatenated MSA. (**b**)The bootstrap value is measured in entire tree and clade levels. The bootstrap of the entire tree is the fraction of trees with an identical topology among bootstrap replicates. The bootstrap value of a clade is the fraction of its corresponding species partition among bootstrap replicated trees. (**c**) We measure discrimination power of bootstrap according to two loss functions. All-or-nothing loss function would assign a loss of 1 or 0 if the inference tree is not identical or identical to the Yeast ToL, respectively. Per Branch loss function assigns a penalty for each clade in the true tree that is missing and another penalty for each clade in the inference tree that is not present in the true tree, typical the Robinson-Foulds metric

The bootstrap of the tree is measured at two different levels: the entire topology or a clade only (Fig. 1b). The bootstrap of the entire tree, *T*, is measured by counting the fraction of replicate set, *R*, topologically identical to *T* [Equation (1), Fig. 1b.2 follow Wong’s methodology].

(1) $$\mathrm{Bootstrap}(T)=\frac{\left|T^{\prime }=T,T^{\prime }\in R\right|}{\left|R\right|}.$$

where *T′* is a replicate from the set *R*.

**Fig. 2.**
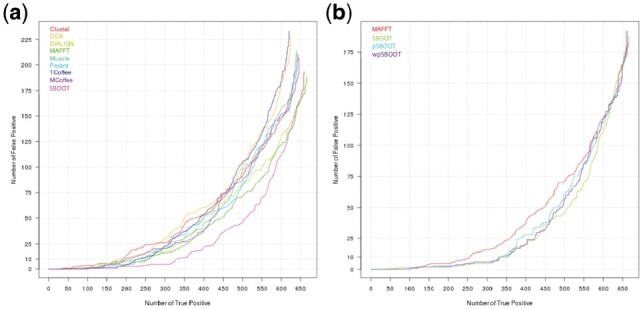
True Positive versus False Positive. (**a**) The horizontal axis indicates the number of trees yielding a yeast ToL topology when ranking 853 selected Wong’s trees by bootstrap values (high to low). The vertical axis shows the number of trees reporting a non-ToL topology. Colored lines correspond to different single aligners. (**b**) Similar analysis focused on SBOOT variations

The bootstrap of a clade (a branch), *C*, is measured as the fraction of its corresponding taxon partition among bootstrap replicated trees [Equation (2), Fig. 1b.1].

(2) $$\mathrm{Bootstrap}(C)=\frac{\left|P\left(C\right)=P\left(C^{\prime }\right),\;C^{\prime }∼\;T^{\prime }\in R\right|}{\left|R\right|}$$

where *C′* is an internal branch of one replicate *T ′* from *R*, *P* represents a partition given by a branch. Take the branch, *c*, of inferred tree as an example, it separates species into two sets (*Saccharomyces cerevisiae*, *Saccharomyces kluyveri*) and *Saccharomyces paradoxus*. Then, we count the proportion of replicates support the partition (branches marked in bold).

### 2.2 Super-MSA alternative bootstrap sampling

The Super-MSA refers to the concatenation of alternative MSAs of the same sequences by reference to the super-matrix procedure of Delsuc (Delsuc *et al.*, 2005) (typically the seven aligners of Wong’s study: ClustalW, Dca, Dialign2, Mafft, Muscle, ProbCons and T-Coffee). In parallel, Ashkenazy *et al.* also names concatenating alternative MSAs as Super-MSA (Ashkenazy *et al.*, 2018). The tree is inferred from the concatenated Super-MSA. We have implemented three sampling procedures to calculate bootstrap supports of the inferred tree. The first one—Super Bootstrap (SBOOT)—involves sampling in each replicate a total number of columns equal to the full length of the concatenated alternative MSAs (Fig. 1a.1, after concatenating seven aligners, the length of Super-MSA is 1050. Then, the length of each replicate is 1050, as well). SBOOT simply applies standard bootstrap on the Super-MSA. However, columns cross the Super-MSA are not independent because of alignments from the same input. Therefore, SBOOT is against bootstrap assumption, columns drawn independently and identically distributed (Felsenstein, 1985). To reduce this bias, we designed two additional protocols, partial SBOOT (pSBOOT) and its weighted version, Weighted Partial SBOOT (wpSBOOT). pSBOOT involves generating replicates having a length equal to the average length of the alternative MSAs (Fig. 1a.2, if the average alignment length of seven aligners is 150, then we sample 150 columns for each replicate instead of 1050. For compromising lost information by partial sampling, we generate seven times more replicates, 700, to have the same amount information with SBOOT). wpSBOOT considers the high of correlation existing between some methods that tend to produce similar or identical MSAs. Under this scheme, the probability of drawing a column from an MSA is inversely proportional to its average similarity with other alternatives (Fig. 1a.3), the sampling weight of the MSA, MSA_*x*_, is obtained using the following formula:

(3) $$\mathrm{SampleWei}\left({\text{MSA}}_{x}\right)=100-\frac{\sum_{y\neq x}^{N}C\mathrm{olumnSim}({\text{MSA}}_{x},{\text{MSA}}_{y})}{N-1}$$

where *N* is the set of concatenated MSAs, MSA_*y*_ is an MSA from the set *N* expect to MSA_*x*_ and *ColumnSim* is the percentage of columns in MSA_*x*_ that are found identically aligned within MSA_*y*_, as returned by the *aln_compare* function of T-Coffee (Taly *et al.*, 2011). The weights are normalized to sum up to 100 for avoiding zero-weight of all identical alignments. The resulting weight is then used when drawing replicates from the concatenated dataset, with each column having a probability to be selected proportional to the weight of the MSA it comes from. According to this weighting scheme, an alignment program that produces alignments similar to others programs will contribute proportionally less to the bootstrap value although this downweighting will be compensated by the presence of many similar MSAs. In contrast, a program that produces disparate alignment has more impact in generating replicates such that they are less similar with the Super-MSA, i.e. lowering bootstrap value in the end.

**Fig. 3.**
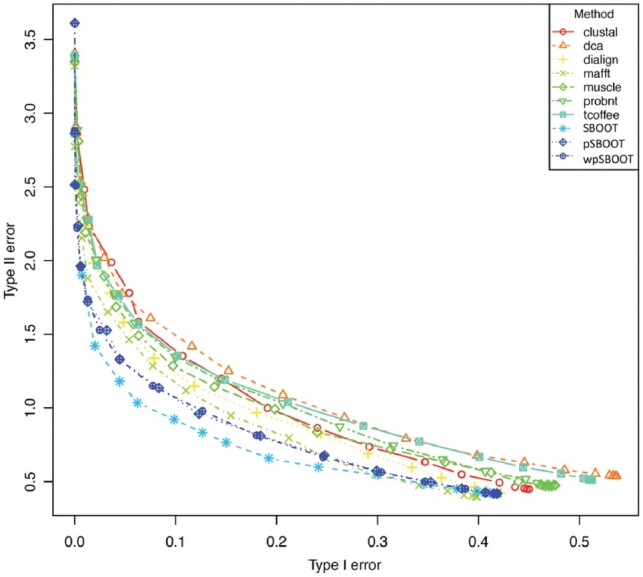
Type I versus Type II error distribution regarding to different bootstrap thresholds on empirical Yeast dataset. Type I error (incorrect clades, *ē*_1_) and Type II error (omitted correct clades, *ē*_2_) of reduced bootstrap trees for each aligner and SBOOT methods

Last but not least, the trees for which support values are being estimated are the same regardless of the evaluation method. They are estimated from the full concatenated set of alternative MSAs. Alternative sampling protocols aim to alter bootstrap values, not to increase tree topology accuracy.

### 2.3 Individual aligners

Phylogenetic reconstructions were evaluated using ClustalW (Thompson *et al.*, 1994), Dca (Stoye, 1998), Dialign2 (Morgenstern, 1999), Mafft (Katoh, 2002), Muscle (Edgar, 2004), ProbCons (Do *et al.*, 2005), T-Coffee (Notredame *et al.*, 2000), PRANK (Löytynoja and Goldman, 2008), SATe (Liu *et al.*, 2009) and M-Coffee (Wallace *et al.*, 2006). M-Coffee and Super-MSA share a dependence on the primary MSAs either used to build T-Coffee library for the consensus MSA or be joined as the concatenated MSA. M-Coffee is a meta-method which combines the outputs of Wong’s seven MSA methods into one single consensus MSA (Wallace *et al.*, 2006). We compare M-Coffee with Super-MSA to investigate two ways for assembling alignment uncertainty, a consensus or a concatenation.

### 2.4 Simulated reference dataset

The simulated datasets used to test Gblocks (Talavera and Castresana, 2007) and trimAl (Capella-Gutiérrez *et al.*, 2009) were combined. They contain three different simulated ROSE-sets (Stoye, 1998), made of datasets having 16, 32 and 64 tips, respectively. Sequence evolutionary rate patterns were extracted from real alignments using TreePuzzle (Schmidt *et al.*, 2002) with an among-site heterogeneity model that assumed a Gamma distribution of 16 rate categories. Then, ROSE created conserved and divergent regions by simulating different positions with above rates and PAM (Dayhoff *et al.*, 1978) evolutionary model. Each set contains 100 datasets generated under a purely asymmetric tree topology (non-ultrametric model with the average and maximum length from the root to the tips, 0.89 and 1.30 substitutions/position; MSA length 400 amino acids). These datasets were aligned with the selected aligners and the resulting models were used to estimate phylogenetic trees using the PROTGAMMAJTT model of RAxML (Stamatakis, 2006) that supports the JTT matrix and the gamma-distributed rate variation.

### 2.5 Empirical reference dataset

We generated an empirical reference phylogenetic dataset, adapted from Wong’s collection (Wong *et al.*, 2008) consisting of 1502 one-to-one orthologous datasets estimated using seven yeast complete genomes with the phylogeny extending back >100 Ma. Each dataset comes along with seven alternative MSAs and their corresponding PAUP Maximum Likelihood tree. It is therefore unclear which fraction of Wong’s datasets can be considered to reflect the Tree of Life (ToL) history. One can nonetheless address this issue by selecting Wong’s collection in datasets more likely to be ToL compliant. We did so by selecting the 853 datasets (Chang *et al.*, 2014) for which at least one of the seven aligners used by Wong yields the established yeast ToL (the complete gene list in Supplementary List).

### 2.6 Topology measure based on loss functions

Holder *et al.* modeled the phylogenetic inference problem under a decision theory framework (Holder *et al.*, 2008) and proposed two loss functions accounting for differences between a tree and its reference. We measure discrimination power of bootstrap according to those two loss functions (Fig. 1c).

- All-or-Nothing loss function: All-or-nothing approach would assign a loss of 1 if the inference tree is not identical to the reference tree and a loss of 0 if it is identical to the true tree. Performances were estimated by the Area Under the Curve (AUC) while using bootstrap values as a predicted confidence with the *ROCR R* package (Sing *et al.*, 2005).
- Per Branch loss function: This function is based on the Robinson–Foulds (RF) metric (Robinson and Foulds, 1981) and penalizes both the splits in the true tree that are missing in the target tree and the clade in the target tree that are missing in the reference (detailed information in Supplementary Section 1).

### 2.7 Topological errors

For investigating the informative value of a bootstrap, we follow the framework by Berry and Gascuel (Berry and Gascuel, 1996). Branches having bootstrap lower than a fixed threshold *S* (i.e. 50 or 95%) were reduced, providing a reduced bootstrap tree ${\overset{-}{t}}_{S}^{*}$ (Berry and Gascuel, 1996). RF is further decomposed into two metrics:

- Type I error (*e_1_*, false positive:FP): number of incorrect clades in the estimate, ${\bar{t}}_{S}^{*}$.
- Type II error (*e_2_*, false negative): number of true clades that are missing from the estimate.

## 3 Results

### 3.1 SBOOT outperforms individual aligners

We first analyzed the relationship between bootstrap support and alignment uncertainty. To that effect, we used the 1502 Wong’s datasets made of seven one-to-one yeast orthologues and binned them by the number of different PAUP Maximum Likelihood tree topologies recovered by the authors. We then used Wong’s methodology to estimate a global bootstrap for each tree and boxed-plotted the distribution by topological bin (Supplementary Fig. S1). This re-analysis of Wong’s data clearly shows an inverse relationship between bootstrap values and aligner’s induced tree topological disagreements. In short, datasets on which aligners disagree tend to have lower bootstrap support, even when this bootstrap is estimated on one aligner at a time. This rather intuitive finding indicates the existence of a relationship between MSA instability (i.e. alternative MSAs yielding different tree topologies) and low bootstrap support.

High bootstrap values might not be direct indicators of phylogenetic accuracy. Sometimes, they reflect the homogeneity of the sampling revealed by the MSA. We first tried to quantify the capacity of bootstrap strategies to discriminate between correct and incorrect topologies. For the All-or-Nothing loss function (Supplementary Table S1), we found individual aligners to be very comparable in terms of overall accuracy. They all manage to reconstruct a similar number of trees having the ToL topology, with MAFFT and PRANK being the most accurate method (665 ToLs, true positive:TP) and T-Coffee the least accurate (620). Noticeably, the concatenated methods do not generate more ToLs than individual aligners (661), i.e. they are not more accurate than single aligners. We were, however, not interested in the overall accuracy but rather in the capacity to discriminate between alternative topologies. To that effect, we sorted the trees by bootstrap values and measured the number of reported TPs (trees having a ToL topology) while accepting 10 or 25 FPs. Results show the individual aligners to be in the same range of accuracy, with an average of 224 TPs for 10 accepted FPs. By contrast, bootstrap values estimated on concatenated versions of these alignments all report more than 300 TPs for the same number of accepted FPs (Fig. 2a). This trend remains similar when considering both the 25 FPs cutoff or the AUC that integrates results over all FP values.

SBOOT outperforms MAFFT (425 versus 359 TPs for 25 FPs) through the sampling strategy across the seven aligners used in Wong’s work (Supplementary Table S1). This improvement comes from the sampling across the different aligners not from sample size (i.e. not from the fact the SBOOT has an increased length with respect to individual aligners). However, the main issue with our SBOOT based bootstrap is the bootstrap value range. While on this dataset, most aligners report bootstrap values in the order of 50% [Supplementary Table S1, column ‘Ave. bootstrap (%)’], SBOOT results in an average bootstrap of 77.31%. This high value is an artifact intrinsic to the drawing procedure where columns are drawn from non-independent MSAs. As a consequence, similar columns are very likely to be over-sampled thus inducing artificially high bootstrap support values.

### 3.2 *pSBOOT* and *wpSBOOT* with compatible bootstrap range

The simplest workaround for this problem is to sample a number of columns similar to the average length of concatenated MSAs, using a partial bootstrapping procedure rather than complete bootstrapping. Originally, it was proposed to unbiasedly estimate confidence level [i.e. *r* will be seven for *N_r_* in the 10th line of the second paragraph, page 51 (Zharkikh and Li, 1995)]. The resulting method (pSBOOT) has an average bootstrap support similar to the one reported on single aligners. It is slightly less discriminative than the full SBOOT but retains a good AUC and a stronger discriminative power than any single aligner alternative methods, especially when accepting only 10 FPs.

Partial bootstrap is not, however, satisfyingly addressing the multiple sampling issue arising when alternative MSAs of the same sequences are concatenated. Its main weakness is its incapacity to account for aligners’ relatedness (i.e. alternative implementation of the same algorithm) and its effect on column over-representation. To addressed this issue, we extended the idea of ClustalW weight scheme, down-weight near-duplicate sequences and up-weight the most divergent ones (Thompson *et al.*, 1994) from sequence level to alignment level. We developed a corrective weighting scheme that up-weights columns emitted by the aligners whose output differs most. Such columns become more likely to contribute when drawing replicates. Results (Supplementary Table S1, Fig. 2b) show a net improvement of the wpSBOOT over most alternative procedures with no significant variation of the bootstrap support values range.

The above All-or-Nothing function penalizes slightly incorrect trees as much as nonsensical trees. Supplementary Table S2 shows an advanced analysis, Per Branch loss function, to distinguish performances between slightly wrong trees and totally nonsense ones. Interestingly, although MAFFT and PRANK are the most effective method in terms of All-or-Nothing loss function, MAFFT performs better than PRANK in Per Branch loss function (Supplementary Table S2, ${ē}_{\mathrm{RF}}$ column). Super-MSA alternative sampling methods do not yield the lowest overall RF score, but however improve the discrimination (Supplementary Table S2, AUC). For instance, the datasets having a wpSBOOT average score higher than 50% are much more topologically correct that datasets selected at the same cutoff with a standard one aligner bootstrap procedure. The distribution of Type I versus Type II error of different thresholds is shown in Figure 3. SBOOT methods show a better balance between Type I and Type II error than single aligners.

### 3.3 Altering of bootstrap support

**Fig. 4.**
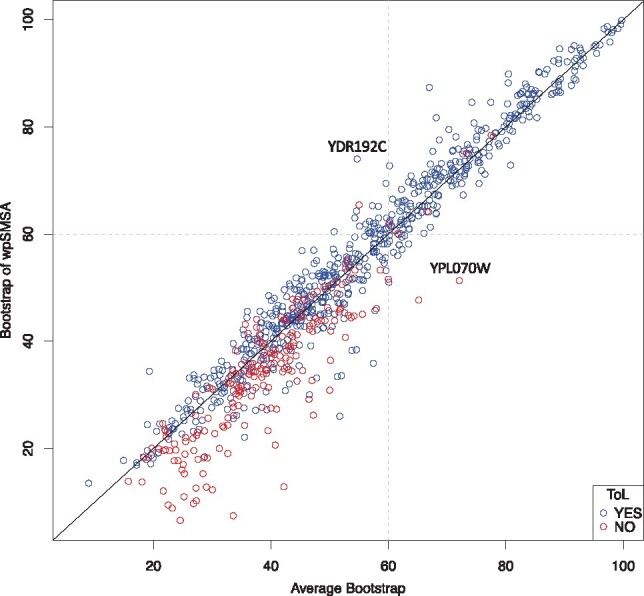
wpSBOOT versus single aligners bootstrap values. Each dot corresponds to one of the 853 datasets extracted from Wong’s data. Blue dots show datasets recapitulating a ToL topology (with wpSBOOT); red dots indicate datasets that do not. The vertical axis corresponds to the wpSBOOT bootstrap value and the horizontal to single aligners’ average bootstrap value

ROC analyses are very useful to obtain an unbiased quantitative estimate of discriminative capacities, but they give little sense of a method usefulness in practical terms. In the case of a bootstrap support measure, the most important is to determine the key threshold value that makes it possible to distinguish between correct and spurious tree topologies. We did this analysis by plotting the respective wpSBOOT bootstrap values on the 853 families (Fig. 4) against similar measures made on the single aligners (average bootstrap). On this plot, the most striking feature is the high level of topological correctness for trees having a bootstrap value higher than 60 (Table 1). Nearly 98% of the 248 trees (=243/248) in this range are topologically correct as opposed to a mere 67% (=382/565) below the 60% bootstrap limit. In the lower range, one can clearly see that correct topologies (in blue) are more often above the main diagonal than below.

**Table 1.** wpSBOOT versus average bootstrap

	wpBS versus singleBS		
	>	=	<
Overall			
wpSBOOT=ToL	383	2	276
wpSBOOT!=ToL	38	1	153
wpBS&aveBS>60			
wpSBOOT=ToL	146	1	96
wpSBOOT!=ToL	3	0	2
wpBS&aveBS<60			
wpSBOOT=ToL	213	1	168
wpSBOOT!=ToL	34	1	148

*Note*: >: number of datasets for which the wpSBOOT bootstrap support values were higher than the average measured on single aligners; =: identical values; <: number of datasets where the values are inferior. Overall, wpSBOOT=ToL: number of datasets for which the wpSBOOT topologies are identical to the ToL. Overall, wpSBOOT!=ToL: number of datasets for which the wpSBOOT topologies are different from the ToL. wpBS&aveBS>60: similar measures restricted to datasets having a bootstrap support higher than 60, with both methods. wpBS&aveBS<60: smaller.

This observation can be quantified (Table 1) and it appears that whenever the wpSBOOT bootstrap is higher than the average bootstrap value measured on single aligners, the resulting tree is 91% [=383/(383 + 38)] likely to be topologically correct as compared to 64% [=276/(276 + 153)] when the wpSBOOT bootstrap support is lower than single aligners. This observation suggests that there exists a very informative relationship between the individual MSA bootstrap readouts and their concatenated counterpart, with the confrontation of these two quantities yielding the most informative bootstrap support analysis reported here.

**Fig. 5.**
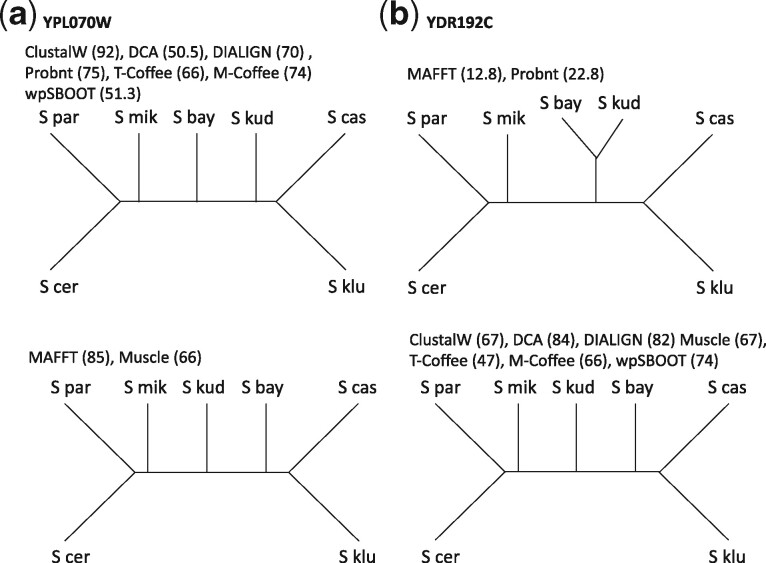
Bootstrap of (a) YPL070W and (b) YDR192C by standard sampling on individual aligners and wpSBOOT. (**a**) There are two topology groups of YPL070W: one by Clustal, DCA, DIALIGN, Probnt, T-Coffee, M-Coffee and Super-MSA; other one by MAFFT and Muscle. (**b**) There are two topology groups of YDR192C: one by MAFFT and Probnt; other one by Clustal, DCA, DIALIGN, Muscle, T-Coffee, M-Coffee and Super-MSA. The bottom two topologies are identical to the Yeast ToL (correct). The number in brackets is corresponding bootstrap support. Tree topology is originally drawn by Phylodendron and is redrawn for fitting page size

Striking examples are YPL070W and YDR192C (Fig. 5). There are two topology groups of YPL070W: a wrong one by Clustal, DCA, DIALIGN, Probnt, T-Coffee, M-Coffee and Super-MSA; and the correct one by MAFFT and Muscle. It is hard to tell whether topology is accurate or not based on standard bootstrap. For example, the bootstrap of ClustalW is quite high, 92, but its topology is wrong. On the other hand, the bootstrap of Muscle is low, 66, but its topology is correct. The bootstrap of wpSBOOT is dramatically decreased from 72.1 (average bootstrap) to 51.3 when its topology is wrong. As alternative, the wpSBOOT bootstrap of YDR192C increases from 54.7 (average bootstrap) to 74 when its topology is correct. In comparison, there could be low bootstrap by individual aligners even their topologies are correct (i.e. T-Coffee: 47, ClustalW and Muscle: 67, Fig. 5b). After incorporating alignment uncertainty into bootstrap sample, our protocols can alter bootstrap value to improve its desirability in tree topology accuracy.

We finally used this dataset to ask about the most relevant combination of methods (Supplementary Fig. S2). Our results show that on the seven considered aligners, similar results are obtained by combining any set of four or more aligners.

### 3.4 Lower Type I error in simulated datasets

This empirical analysis has two main limitations: it depends on an *ad-hoc* benchmark in which the exact fraction of ToL compliant datasets is an unknown quantity. Secondly, individual datasets of seven taxa are not challenging enough to extrapolate these results to real life phylogenies. For this reason, we decided to complete our analysis with larger simulated datasets of up to 64 taxa. This larger dataset gave us a chance to estimate branch support rather than full tree support. Such an analysis is of more practical relevance since trees are often used on a branch-by-branch basis, using bootstrap support to identify unresolved nodes. Table 2 shows that wpSBOOT is consistently one of the methods returning the largest fraction of topologically correct trees. Its level of accuracy is comparable to SATe, a state-of-the-art phylogeny aware aligner. The most important observation on this table is certainly the good scaling capacities of wpSBOOT. For instance, when accepting 100 FP nodes, wpSBOOT manages to recover more correct branches than any alternative method tested here. By this criterion and considering the three datasets at four cutoff levels (10, 25, 50 and 100 FPs), wpSBOOT ranks #1 on 7 out of 12 tests and is among the two best (usually with SATe) on 3 out of 12. By these measures wpSBOOT is therefore the most trustworthy bootstrap support evaluation method tested here. Table 3 shows that wpSBOOT is also a consistently one of the methods returning the lowest RF score. When considering subset of highly supported trees, wpSBOOT has not only lower RF but also lower Type I error, meaning less false positive—an important practical property. The distribution of Type I versus Type II of reduced trees on different bootstraps is shown in Supplementary Figure S3.

**Table 2.** The performances of All-or-Nothing loss function on simulated datasets

			TPs				
Aligner	AveBoot(%)	AUC	10FP	25FP	50FP	100FP	Total
16tips							1300
ClustalW	83.5	0.909	883	1020	1096	1165	1180
MAFFT	82.8	0.912	**898**	1019	1095	1168	1178
ProbCons	82.5	**0.916**	854	993	1088	1159	1172
PRANK	83.5	0.901	812	967	1059	1121	1162
SATe	83.4	0.910	854	1000	1097	1166	1179
wpSBOOT	83.4	0.903	854	**1047**	**1099**	**1177**	**1186**
32tips							2900
ClustalW	54.3	0.805	267	395	532	705	1547
MAFFT	55.0	0.843	579	782	907	1091	1834
ProbCons	56.4	0.841	**680**	818	923	1121	1882
PRANK	58.7	0.803	192	399	582	774	1612
SATe	57.3	**0.852**	627	829	**1033**	1179	1918
wpSBOOT	56.6	0.843	669	**883**	1007	**1225**	**1944**
64tips							6100
ClustalW	47.4	0.801	276	424	636	883	2843
MAFFT	48.4	0.819	523	806	1048	1322	3262
ProbCons	49.6	0.818	872	1134	1350	1533	3462
PRANK	52.8	**0.832**	257	430	632	916	2680
SATe	51.5	0.815	1000	**1269**	**1433**	1539	**3544**
wpSBOOT	49.8	0.828	**1026**	1203	1360	**1703**	3514

*Note*: Aligner: considered aligners. Note that wpSBOOT represents the concatenation of ClustalW, MAFFT, ProbCons, PRANK and SATe MSAs. AveBoot(%): average bootstrap support measured on the simulated dataset. AUC: area under the ROC curve of the corresponding aligner. 10FP: number of nodes defining target split properly recovered when accepting 10 incorrect splits on a list of splits ranked by bootstrap support. 25, 50, 100FP: similar. Total: total number of nodes on the considered datasets (16, 32 and 64 tips lines) or total number of correctly recovered nodes. The three sections labeled 16, 32 and 64 tips correspond to similar analyses done on datasets of different sizes. Best performance in each column is marked in bold.

**Table 3.** The performances of Per Branch loss function on simulated datasets—accuracy of estimated trees for the standard Robinson and Foulds distance

	Ave. boot. (%)	$\hat{t}$		${t¯}_{50\%}^{*}$			${t¯}_{95\%}^{*}$		
Aligner		${ē}_{\mathrm{RF}}$	${ē}_{1}={ē}_{2}$	${ē}_{\text{RF}1}$	${ē}_{1}$	${ē}_{2}$	$e_{\mathrm{RF}}$	${ē}_{1}$	${ē}_{2}$
16tips									1300
ClustalW	83.52	0.185	0.092	0.192	0.058	0.135	0.598	0.000	0.598
MAFFT	82.80	0.188	0.094	0.188	0.055	0.134	0.613	0.004	0.609
ProbCons	82.48	0.197	0.098	0.196	0.058	0.138	0.611	0.002	0.609
PRANK	83.38	0.212	0.106	0.216	0.070	0.145	0.588	0.000	0.588
SATe	83.36	0.186	0.093	**0.186**	0.057	**0.128**	0.593	**0.000**	0.593
wpSBOOT	83.43	**0.175**	**0.088**	**0.186**	**0.054**	0.132	**0.584**	0.002	**0.582**
32tips									2900
ClustalW	54.26	0.933	0.467	0.850	0.253	0.597	0.923	0.051	0.872
MAFFT	55.00	0.735	0.368	0.673	0.143	0.530	0.862	0.014	0.849
ProbCons	56.38	0.702	0.351	0.655	0.145	0.510	0.847	0.004	0.843
PRANK	58.73	0.888	0.444	0.835	0.270	0.565	0.894	0.063	0.831
SATe	57.28	0.677	0.339	**0.619**	0.128	**0.491**	**0.843**	0.006	**0.837**
wpSBOOT	56.62	**0.659**	**0.330**	0.623	**0.118**	0.505	0.849	**0.004**	0.844
64tips									6100
ClustalW	47.38	1.068	0.534	0.956	0.273	0.683	0.985	0.039	0.947
MAFFT	48.39	0.930	0.465	0.838	0.189	0.650	0.946	0.014	0.932
ProbCons	49.60	0.865	0.432	0.789	0.165	0.624	0.935	0.002	0.933
PRANK	52.84	1.121	0.561	0.980	0.323	0.657	0.971	0.055	0.916
SATe	51.49	**0.838**	**0.419**	0.772	0.168	**0.604**	**0.928**	0.002	**0.926**
wpSBOOT	49.77	0.848	0.424	**0.764**	**0.146**	0.618	**0.928**	**0.000**	0.928

*Note*: The average strand (*λ *=1) Robinson and Foulds error, ${\bar{e}}_{\mathrm{RF}}$, induced by the original tree, $\hat{t}$, and by reduced bootstrap tree, ${\bar{t}}_{50\%}^{*}$ and ${\bar{t}}_{95\%}^{*}$. This error is decomposed into Type I error (incorrect clades) and Type II error (omitted correct clades), denoted ${\bar{e}}_{1}$ and ${\bar{e}}_{2}$, respectively. Best performance in each column is marked in bold.

## 4 Conclusion and discussion

In this paper, we describe a novel approach able to incorporate MSA uncertainty within phylogenetic trees bootstrap support values. Our method simply requires concatenating several alternative MSAs of the considered sequences into a Super-MSA. This SBOOT is then used to draw bootstrap replicates. We describe and validate two slight variations around this protocol: a partial sampling meant to avoid re-sampling and another one combining partial sampling with a weighting scheme, to incorporate individual MSAs information content. We validated the discriminative power of this new bootstrap-based confidence measure and found it to be more discriminative than similar measures made on single MSAs. We confirmed this observation on both empirical and simulated datasets. The simulated datasets also made it possible to show that this approach scales better than standard bootstrap support measures and is useful both at the full topology level and at the split (node) level when estimating phylogenetic trees confidence using maximum likelihood.

This methodology is important because it addresses a key problem when doing phylogenetic reconstruction: MSAs are not data, they are models. For a long time, phylogenetic textbooks have all but ignored this reality, with MSAs always appearing as idealized ungapped blocks sprinkled with a few strategic mutations. In practice, however, it is as difficult to estimate an MSA, as it is to estimate an evolutionary tree. As a consequence, most evolutionary models combine three kinds of noise: the one resulting from approximate MSAs, the one resulting from uneven evolutionary sampling, and the one resulting from partial ML optimization when estimating the tree. Until now, the MSA induced noise was missing from bootstrap-based support estimates. We show here that re-integrating this source of information is relatively easy and extremely informative to accurately assess tree reliability.

Our approach is very generic. We show that any reasonable amount of MSA sampling can help improve bootstrap support. The use of seven hand-picked aligners may seem arbitrary, but in our view, it shows that the SBOOT bootstrap is most likely insensitive to the nature of combined aligners, provided they bring enough diversity. One could easily replace the aligners by exploring the parameter space (gap penalties) or by exploring the tiebreak space (PRANK replicates). We expect such approaches to be especially fruitful when dealing with nucleic acids MSAs that tend to be less stable than their protein counterparts, especially when no constraint exists on secondary structure conservation. Recently, Lemoine *et al.* establish a new bootstrap version based on transfer distance, which overcomes the low support problem of the original bootstrap in large scale data (Lemoine *et al.*, 2018). Applying our alternative sampling approach on the new bootstrap will be investigated in the further. Ashkenazy *et al.* found the concatenation of alternative MSAs from GUIDENCE2 can improve tree topology accuracy (Ashkenazy *et al.*, 2018). Another further extension is to apply alternative bootstrap on GUIDENCE2 Super-MSA. We expect enriching evolutionary signal not only tree topology but also bootstrap discrimination power. Overall, the SBOOT should provide a good correction of well-known MSA reconstruction artifacts such as dynamic programing tiebreaks or guide tree influence (Löytynoja and Goldman, 2009). In an ideal world, all alternative MSAs would be used and weighted using some informative posterior probability. A possible model can be the approximately unbiased (AU) test by multi-scale bootstrap technique (Shimodaira, 2002). pSBOOT is adapted from the idea of Zharkikh and Li’s work (Zharkikh and Li, 1995), which has been shown similar to AU test (Shimodaira, 2002). Both ZL and AU tests aim to estimate unbiased *P*-value by bootstrap values from different scales. Our approach is one special case of multi-scale bootstrap for *r*_1__ _= 0.14 and *K *=* *1. However, we do not use the information of bootstrap values from different scales (*K *≥* *2). In the future, AU test should be extended under a situation, dependent sequences. It will give an idea about how devise alignments should be concatenated to have an unbiased *P*-value estimation. Such an estimate remains, however, challenging (Blackburne and Whelan, 2013) leaving the problem of quantifying tree uncertainty rather open (Redelings and Suchard, 2005).

## Supplementary Material

btz082_Supplementary_Data
