# Supplementary material for: Incorporating alignment uncertainty into Felsenstein’s phylogenetic bootstrap to improve its reliability
Source: Bioinformatics. 2019 Feb 6;37(11):1506–14. doi: 10.1093/bioinformatics/btz082 (PMC8275982; doi:10.1093/bioinformatics/btz082)
Supplement: btz082_Supplementary_Data [file btz082_supplementary_data.zip › SupDoc.pdf]

Supplemental Document

Incorporating alignment uncertainty into Felsenstein's phylogenetic bootstrap to improve its reliability

Supplemental Section 1

When considering 64 tips topologies, it is common for all the alignment methods to report a tree topologically different from the reference. For this reason, we used the correctness of internal nodes as a measure of success. Internal branches of a given tree were labeled as proven positives when their split matched the reference (as estimated using "Fast Tree-Comparison Tools" (Price *et al.*, 2009)) and proven negative otherwise. Branches were ranked by their bootstrap supports in order to perform a Receiving Operator Characteristic (ROC) curve validation and estimate the Area Under the Curve (AUC) with the *ROCR R* package (Sing *et al.*, 2005).

For each of these 853 datasets, we estimated seven alternative PAUP ML trees from the original MSAs produced by the seven alternative aligners. Performances were estimated by doing a ROC analysis on the resulting trees with topologically correct trees (i.e. identical to the ToL) labeled as Proven Positives and the others as Proven Negatives while using bootstrap values of entire tree as a ranking score.

Reference:

Price,M.N. *et al.* (2009) Fasttree: Computing large minimum evolution trees with profiles instead of a distance matrix. *Mol. Biol. Evol.*, **26**, 1641–1650.

Sing,T. *et al.* (2005) ROCR: Visualizing classifier performance in R. *Bioinformatics*, **21**, 3940–3941.

Supplemental Figures

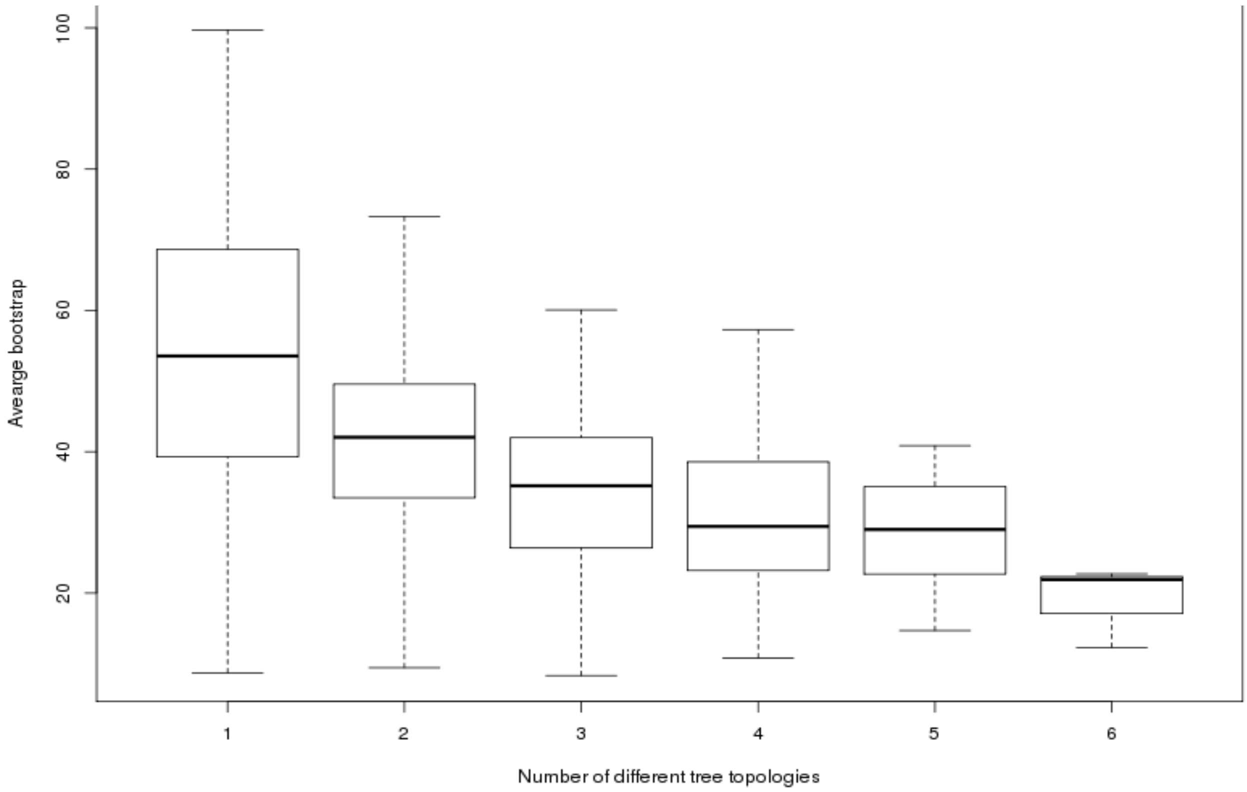

Fig. 1. Relationship between bootstrap values and topological stability. Each boxplot represents the distribution of bootstrap values. The *x*-axis represents the number of different tree topologies reported by 7 aligners on identical datasets using Wong's 1502 yeast 1-to-1 orthologous collections. There is no case with seven different topologies.

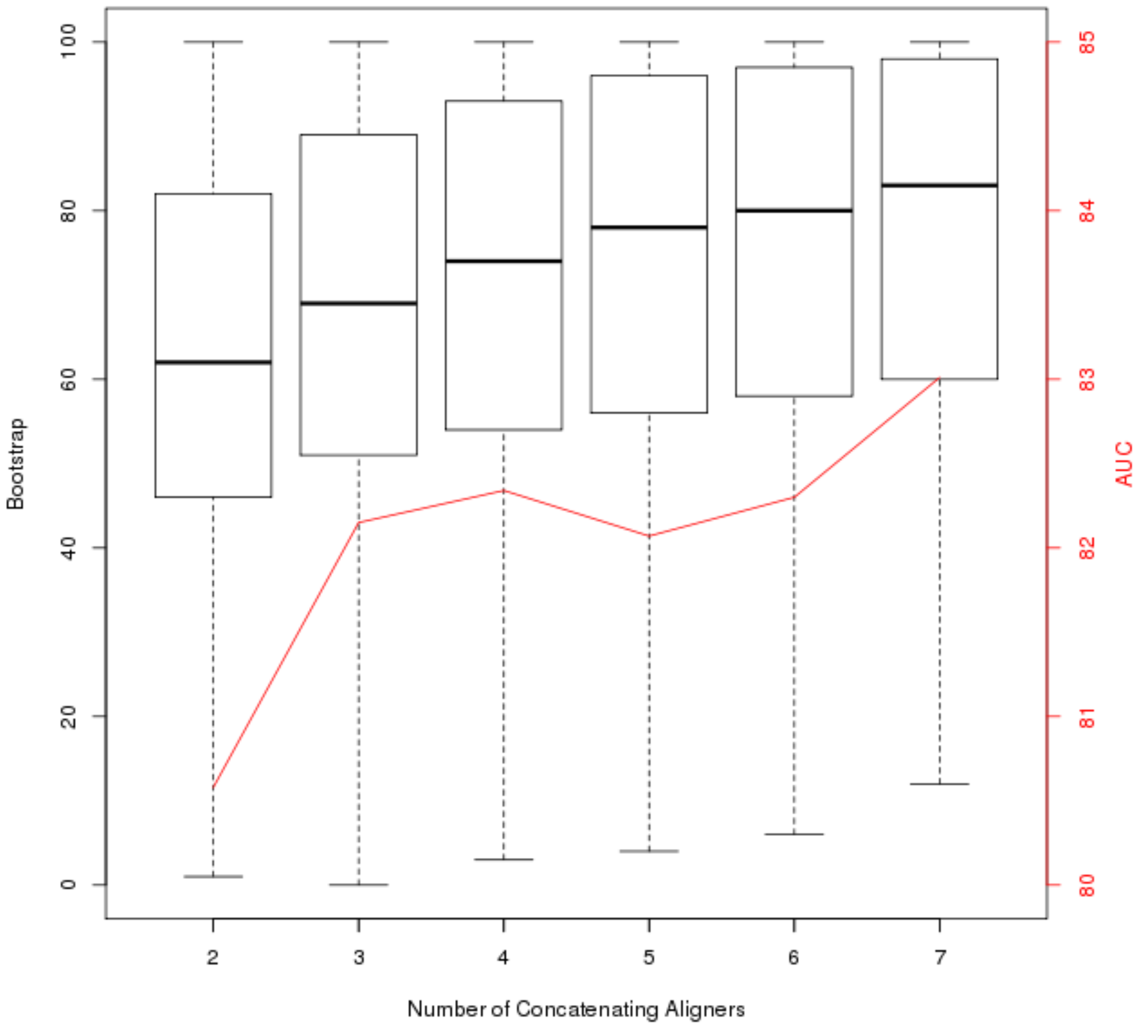

Fig. 2. Effect of aligners number when concatenating. Boxplots show the variation in bootstrap support; the horizontal axis shows the number of considered aligners. The red curve shows the average Area Under the Curve (%) corresponding to each combination. The reported values correspond to an exhaustive exploration of all possible C_x^7 combinations, where *x* is the number of considered aligners.

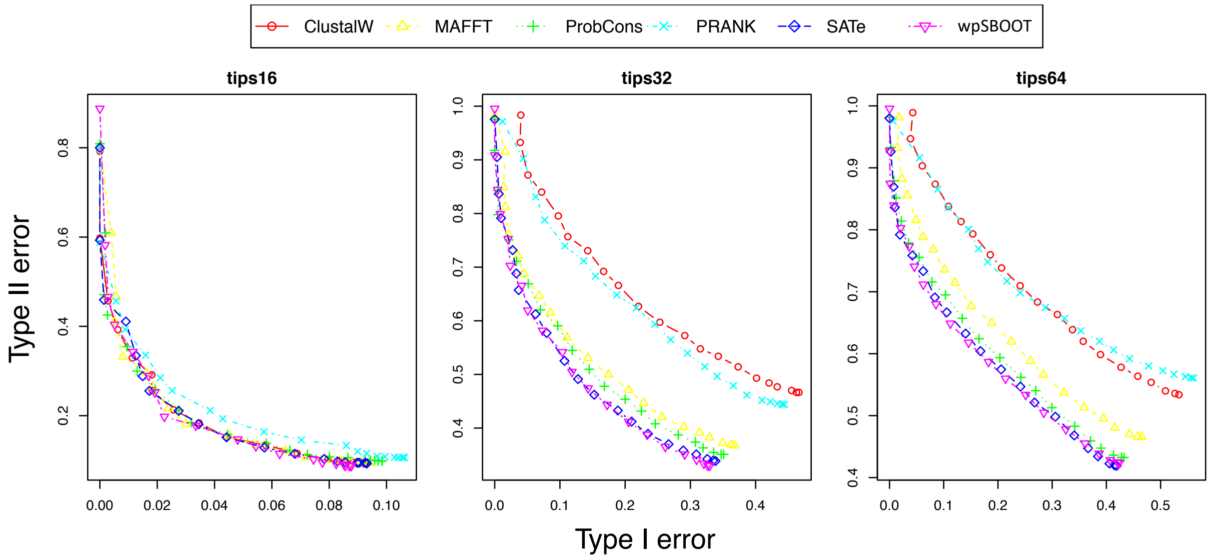

Fig. 3. Type I versus Type II error distribution regarding to different bootstrap thresholds on simulated dataset. Type I error (incorrect clades, $\bar{e}_1$) and Type II error (omitted correct clades, $\bar{e}_2$) of reduced bootstrap trees for each aligner and wpSBOOT.

Supplemental Tables

Table 1. All-or-Nothing loss function - Quantitative comparison of bootstrap support usefulness across aligners. *aligner*: selected aligner. *Ave bootstrap*: average bootstrap value as defined by Wong. *AUC*: Area Under the Curve measured by the ROC analysis. *10 FPs*: number of True Positives returned for 10 False Positives. *25 FPs*: idem. *total*: total number of trees with a Tree of Life topology. Best performance in each column is marked in bold.

			TPs		
Aligner	Ave. bootstrap (%)	AUC	10 FPs	25 FPs	total
ClustalW	51.31	0.7521	185	274	643
DCA	50.62	0.7694	194	284	624
DIALIGN	51.94	0.7618	253	340	659
MAFFT	52.82	0.7750	253	359	**665**
Muscle	52.35	0.7771	224	315	639
Probnt	50.96	0.7790	256	312	642
T-Coffee	51.21	0.7889	234	311	620
M-Coffee	51.41	0.7688	193	325	646
PRANK	53.92	0.7706	243	325	**665**
SATe	53.25	0.8074	313	395	659
SBOOT	77.31	**0.8301**	329	**425**	661
pSBOOT	50.96	0.8140	342	385	661
wpSBOOT	50.86	0.8215	**353**	423	661

Table 2. Per Branch loss function, Type I and Type II error of the original tree and induced trees. Best performance in each column is marked in bold.

Aligner	AUC	$\hat{t}$		$\bar{t}^*_{50\%}$			$\bar{t}^*_{95\%}$		
		$\bar{e}_{RF}$	$\bar{e}_1 = \bar{e}_2$	$\bar{e}_{RF}$	$\bar{e}_1$	$\bar{e}_2$	$\bar{e}_{RF}$	$\bar{e}_1$	$\bar{e}_2$
ClustalW	99.31%	0.900	0.450	1.106	0.240	0.865	2.812	0.002	2.810
DCA	98.91%	1.074	0.537	1.202	0.267	0.934	2.876	0.001	2.875
DIALIGN	99.43%	0.844	0.422	1.068	0.244	0.824	2.798	0.001	2.797
MAFFT	99.12%	**0.797**	**0.399**	1.009	0.212	0.797	2.729	0.002	2.727
Muscle	99.68%	0.952	0.476	1.076	0.240	0.836	2.761	0.004	2.757
Probnt	99.63%	0.945	0.472	1.137	0.263	0.875	2.849	0.002	2.846
T-Coffee	99.34%	1.025	0.512	1.164	0.286	0.878	2.822	0.000	2.822
M-Coffee	99.24%	0.947	0.474	1.098	0.236	0.863	2.805	0.001	2.804
PRANK	88.05%	1.556	0.778	1.682	0.573	1.108	3.219	0.263	2.955
SATe	99.27%	0.857	0.428	0.995	0.218	0.777	2.763	**0.000**	2.763
SBOOT	99.81%	0.837	0.419	**0.831**	0.346	**0.485**	**1.401**	0.022	**1.379**
pSBOOT	99.78%	0.837	0.419	0.995	0.184	0.811	2.855	**0.000**	2.855
wpSBOOT	**99.84%**	0.837	0.419	0.995	**0.181**	0.815	2.871	**0.000**	2.871

The average strand Robinson and Foulds error, $\bar{e}_{RF}$, induced by the original tree, $\hat{t}$, and by reduced bootstrap tree, $\bar{t}^*_{50\%}$ and $\bar{t}^*_{95\%}$. This error is decomposed into Type I error (incorrect clades) and Type II error (omitted correct clades), denoted $\bar{e}_1$ and $\bar{e}_2$, respectively.
